# Laparoscopic intraperitoneal onlay mesh (IPOM) repair for lumbar hernia: a case series of three patients with review of literature

**DOI:** 10.1097/RC9.0000000000001162

**Published:** 2026-10-21

**Authors:** Md Anas, Imad Ali, Shivendu Purushottam

**Affiliations:** Department of Surgery, Jawaharlal Nehru Medical College, AMU, Aligarh, India

**Keywords:** lumbar hernia, Grynfeltt-Lesshaft hernia, Petit’s hernia, incisional hernia, laparoscopic intraperitoneal onlay mesh (IPOM) repair, case series

## Abstract

**Introduction:**

Lumbar hernias are uncommon posterolateral abdominal wall defects that present either spontaneously (primary) or as acquired (secondary) hernias. Their rarity and non-specific clinical presentation often lead to delayed diagnosis and management. Laparoscopic intraperitoneal onlay mesh (IPOM) repair offers wide intra-abdominal visualization and tension-free prosthetic reinforcement, but the supporting evidence is limited to case reports and small series.

**Presentation of cases:**

This was a prospective, single-center case series of three consecutive patients diagnosed with a lumbar hernia at a tertiary care center in Northern India between January 2025 and December 2025. Case 1 involved an 18-year-old female with chronic right iliac fossa pain who was initially suspected to have chronic appendicitis or abdominal tuberculosis, in whom a primary lumbar hernia was diagnosed during diagnostic laparoscopy. Cases 2 and 3 involved a 55-year-old female and a 30-year-old male who presented with acquired incisional lumbar hernias following previous nephrectomy and open pyelolithotomy, respectively. All three patients underwent successful IPOM repair with 15 × 15 cm composite mesh placement, fixed with non-absorbable tackers. There were no intraoperative or postoperative complications. All patients were discharged in satisfactory condition with no recurrence or major complaints at follow-up.

**Conclusion:**

Laparoscopic IPOM repair is feasible and associated with good postoperative recovery for both primary and acquired lumbar hernias. A high index of clinical suspicion is essential, particularly in patients with unexplained flank pain or a history of retroperitoneal surgery.

## Introduction

Lumbar hernias are rare posterior abdominal wall defects, accounting for approximately 1.5% of all abdominal hernias^[^1,2^]^. They occur through anatomical weaknesses in the lumbar region, most commonly the superior lumbar triangle (Grynfeltt-Lesshaft space), bounded by the 12th rib, the paraspinal muscles, and the internal oblique, or the inferior lumbar triangle (Petit’s space), bounded by the iliac crest, the latissimus dorsi, and the external oblique^[^3,4^]^.

HIGHLIGHTS

- Lumbar hernias are rare posterior abdominal wall defects that occur through anatomical weaknesses in the lumbar region.
- Lumbar hernias are frequently missed and diagnosed late because their presentation mimics other flank pathologies, and the bulky lumbar muscles cover the hernial defect.
- Primary lumbar hernias may present atypically, mimicking acute appendicitis.
- Laparoscopic intraperitoneal onlay mesh repair is feasible and associated with favorable short- to intermediate-term outcomes for lumbar hernias.

Lumbar hernias are divided into primary and acquired (secondary) hernias. Primary hernias (congenital or spontaneous) account for roughly 20% of all cases and develop from a combination of congenital fascial weakness, progressive muscular atrophy with age, and the mechanical effects of prolonged, raised intra-abdominal pressure^[^5^]^. The remaining 80% are acquired and are predominantly iatrogenic following flank incisions for urological or orthopedic surgeries, post-traumatic, or, rarely, infection-induced^[^6,7^]^.

The bulky overlying musculature masks the hernia, presenting as vague flank pain that is easily misdiagnosed. Patients may present with non-specific back pain, or hernias may be mistakenly identified as subcutaneous lipomas, hematomas, or abscesses ^[^7,8^]^.

Once diagnosed, repair is generally advised because the reported incarceration risk is up to 30.8%^[^2^]^. Historically, open repair by primary sutures or myofascial flaps is associated with a high recurrence rate, since the lumbar region lacks a strong midline structure for tension-free closure^[^9^]^. Laparoscopic mesh techniques, including transabdominal preperitoneal (TAPP)^[^10^]^, total extraperitoneal (TEP)^[^11^]^, and intraperitoneal onlay mesh (IPOM)^[^12^]^, have improved outcomes considerably for both primary and acquired lumbar hernias.

Lumbar hernias are uncommon, and most available literature is limited to case reports or case series^[^13^]^. We present three cases of lumbar hernia – one primary hernia discovered incidentally during diagnostic laparoscopy and two acquired hernias following renal surgery – all successfully managed by laparoscopic IPOM repair, with a review of the current literature. This case series has been reported in line with the PROCESS 2025 guidelines^[^14^]^.

## Materials and methods

This study was a prospective case series of three consecutive patients with lumbar hernias who were managed surgically between January and December 2025 at a tertiary care center in Northern India. The primary operating surgeon was an assistant professor with more than 6 years of clinical experience and a fellowship in minimal access surgery.

Inclusion criteria included patients aged ≥ 16 years with a radiologically confirmed diagnosis of a primary or acquired lumbar hernia who were fit for laparoscopic surgery under general anesthesia. Both male and female patients were included. Exclusion criteria included patients with recurrent lumbar hernias, severe comorbidities [American Society of Anesthesiologists (ASA) physical status IV or higher], or those who did not provide consent.

Postoperative follow-up was standardized at 1 month, 6 months, 9 months, and 1 year. Follow-up assessments were conducted solely via physical examination to evaluate for recurrence, chronic pain, seroma, infection, and abdominal wall bulging. Postoperative imaging was not routinely performed to minimize radiation exposure and health care costs, as no patients exhibited clinical signs or symptoms of recurrence or complications. A summary of the cases is given in Table 1.

**Table 1:** Summary of cases.

Parameter	Case 1	Case 2	Case 3
Age/sex	18 yr/F	55 yr/F	30 yr/M
BMI (kg/m^2^)	19.2	26.4	22.8
ASA grade	I	II	I
Type of lumbar hernia	Primary (incidental)	Acquired (post-nephrectomy)	Acquired (post-open pyelolithotomy)
Presenting complaint	Chronic RIF pain	Left lumbar swelling	Left lumbar swelling
Duration	1 year	6 months	4 months
Preceding surgery	None	Left nephrectomy (1 year prior)	Left open pyelolithotomy (9 months prior)
Defect size	4 × 4 cm	6 × 5 cm	5 × 4 cm
Hernial contents	Appendix, caecum	Retroperitoneal fat, descending colon	Retroperitoneal fat, descending colon
Complications	None	None	None
Hospital stay	2 days	4 days	3 days

## Presentation of cases

### Case 1: Incidental primary lumbar hernia mimicking appendicitis

#### Patient information

An 18-year-old female patient presented to the surgical outpatient department with a history of intermittent right iliac fossa (RIF) pain for 1 year, without any comorbidities. There was no history of trauma or surgery.

#### Clinical findings

Physical examination revealed mild tenderness in the RIF without guarding or rigidity. There was no palpable flank mass or swelling. Findings remained unchanged in different positions, during the Valsalva maneuver and with coughing.

#### Diagnostic assessment

Routine blood investigations were within normal limits. An initial abdominal ultrasound followed by a contrast-enhanced computed tomography scan suggested mild inflammatory changes in the appendix and mesenteric lymphadenopathy. A preoperative diagnosis of subacute appendicitis or abdominal tuberculosis was considered.

#### Management

The patient was scheduled for and underwent diagnostic laparoscopy under general anesthesia in the supine position. Pneumoperitoneum was created with carbon dioxide and maintained at 14 mmHg. Entry was obtained using a closed Veress needle technique at the primary port site, and all subsequent ports were placed under direct vision. A camera port was placed at the left Palmer’s point, with two working ports inserted: one in the left lumbar region (approximately 10 cm lateral to the umbilicus) and another in the hypogastric region (approximately 10 cm below the umbilicus). Intraoperatively, the abdominal cavity revealed an unexpected defect measuring 4 × 4 cm in the right posterolateral abdominal wall, as shown in Figure. 1A, consistent with a primary inferior lumbar (Petit’s) hernia containing the appendix and cecum. However, the appendix appeared normal and was not removed. Given the symptomatic nature of her right-sided pain, the decision was made to repair the hernia. The sac was reduced, and the fascial defect could not be closed primarily because the edges could not be approximated. A 15 × 15 cm composite mesh was then placed intraperitoneally with generous overlap, fixed using non-absorbable tackers, and the margins were sutured with Prolene, as shown in Figure 1B. There were no intraoperative complications and minimal blood loss.

**Figure 1.**
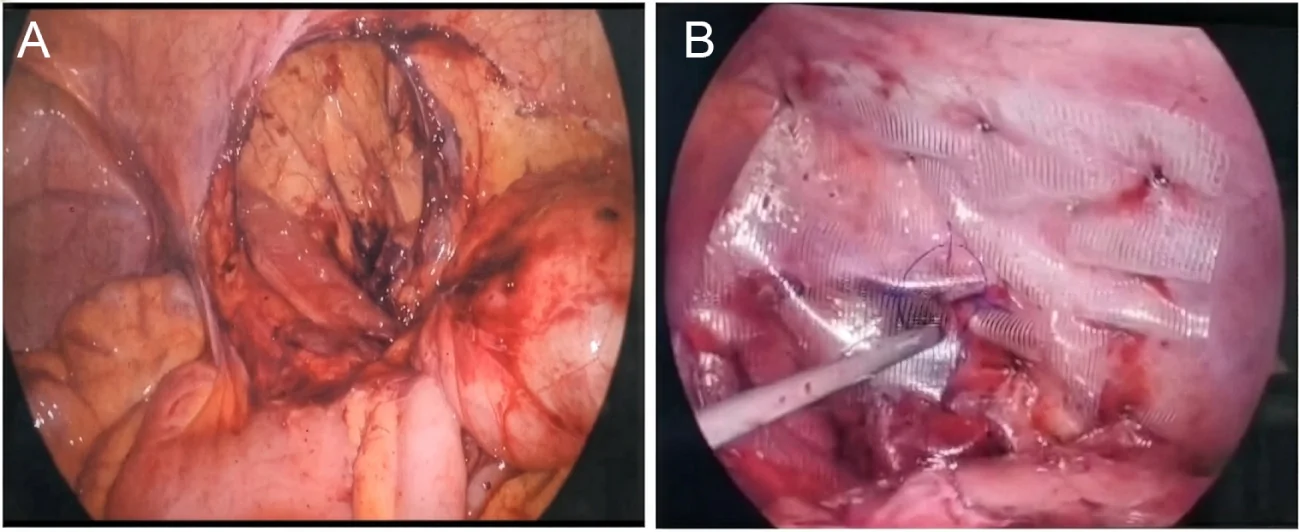
(A) A 4 × 4 cm defect in the right posterolateral abdominal wall. (B) Composite mesh fixation with a tacker.

#### Follow-up and outcome

The patient had an uneventful postoperative recovery. She was mobilized on the evening of surgery and discharged on postoperative day 2. At the 1-year follow-up, she remained asymptomatic with no evidence of recurrence.

### Case 2: Acquired lumbar hernia post-nephrectomy

#### Patient information

A 55-year-old woman presented with a reducible swelling in the left lumbar region that had been progressively increasing in size over the past 6 months; she had no known comorbidities. She had a history of an open left nephrectomy performed 1 year ago for a non-functional left kidney.

#### Clinical findings

Clinical examination revealed a distinct 6 × 5 cm swelling in the left flank region over her previous surgical scar. There was a positive cough impulse, and the swelling was reducible in the lateral decubitus position.

#### Diagnostic assessment

An abdominal ultrasound confirmed an incisional lumbar hernia measuring 6 × 5 cm in the left posterolateral abdominal wall, containing retroperitoneal fat and the descending colon, without signs of bowel obstruction or ischemia.

#### Management

The patient was planned for and taken for laparoscopic IPOM repair under general anesthesia in the right lateral position. Pneumoperitoneum was created with carbon dioxide and maintained at 14 mmHg. Entry was obtained using a closed Veress needle technique at the primary port site, and all subsequent ports were placed under direct vision. The camera port was placed at the umbilicus, with one working port at the left Palmer’s point and a second working port in the left iliac fossa (approximately 5 cm below and lateral to the umbilicus). By a lateral transabdominal approach, the adherent retroperitoneal fat and the descending colon were dissected free to expose the fascial defect of approximately 6 × 5 cm, as shown in Figure 2A. The defect was primarily closed using Prolene, as shown in Figure 2B. A 15 × 15 cm composite mesh was placed intraperitoneally, covering the defect with wide margins. The mesh was fixed with non-absorbable tackers. No intraoperative complications were noted, and there was minimal blood loss.

**Figure 2.**
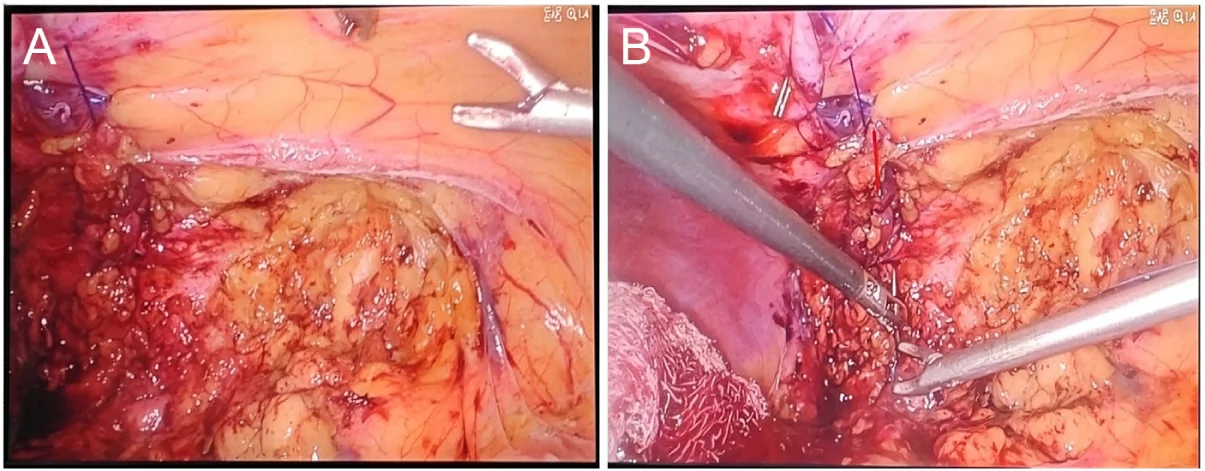
(A) A 6 × 5 cm fascial defect in the left posterolateral wall. B) Primary closure of the defect with Prolene.

#### Follow-up and outcome

The postoperative course was uneventful. The patient was discharged on postoperative day 4. The patient experienced expected mild-to-moderate postoperative surgical-site pain that was effectively managed with standard oral analgesics and was not classified as a complication. At the 1-year follow-up, the patient was in satisfactory condition with no clinical signs of recurrence.

### Case 3: Acquired lumbar hernia post-open pyelolithotomy

#### Patient information

A 30-year-old male manual laborer presented with left lumbar swelling and dull, aching pain for the past 4 months, without any known comorbidities. He had a history of a left open pyelolithotomy 9 months ago.

#### Clinical findings

Examination showed a visible bulge in the left flank protruding through the previous surgical scar. A cough impulse was present, and the swelling was reducible.

#### Diagnostic assessment

Abdominal ultrasound showed a 5 × 4 cm fascial defect in the superior lumbar region, containing retroperitoneal fat and descending colon.

#### Management

The patient also underwent laparoscopic IPOM repair under general anesthesia in the right lateral position. Pneumoperitoneum was created with carbon dioxide and maintained at 14 mmHg. The entry was obtained using the closed Veress needle technique at the primary port site, and all subsequent ports were placed under direct vision. The camera port was placed at the umbilicus, with one working port at the left Palmer’s point and a second working port in the left iliac fossa (approximately 5 cm below and lateral to the umbilicus). Through a lateral transabdominal approach, we mobilized the left colon medially to fully expose the fascial defect measuring approximately 5 × 4 cm, and the hernia contents were reduced without difficulty. The defect was primarily closed with Prolene. The repair was then completed using a 15 × 15 cm composite mesh, which was fixed with non-absorbable tackers, as shown in Figure 3. No intraoperative complications were observed, and there was minimal blood loss.

**Figure 3.**
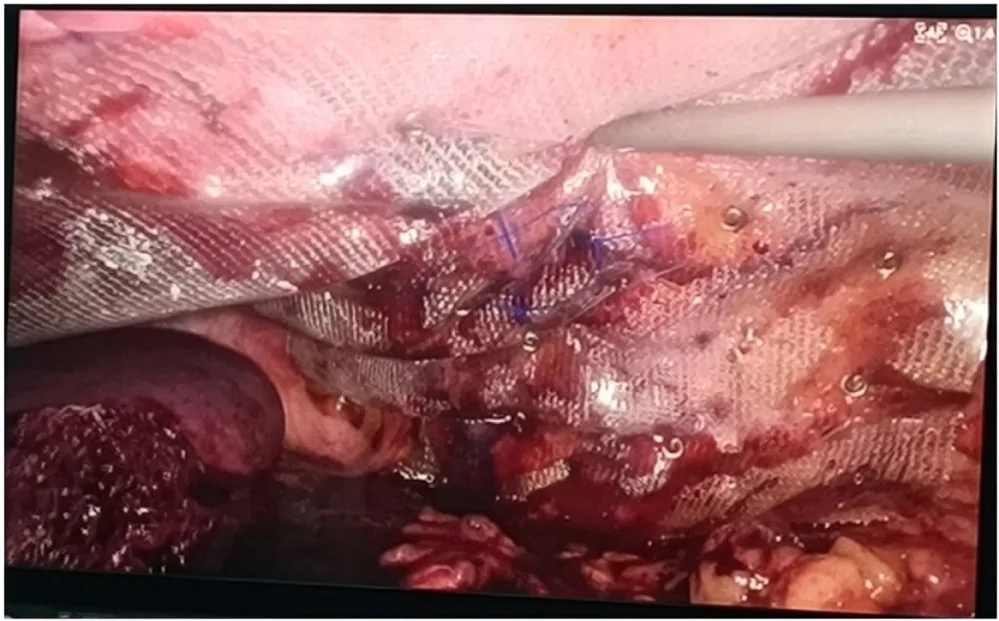
Composite mesh fixation with a tacker.

#### Follow-up and outcome

The patient recovered well and was discharged on postoperative day 3. He was advised to avoid heavy lifting for 12 weeks. He successfully returned to manual labor with no hernia recurrence noted at the 9-month follow-up.

## Discussion

Lumbar hernias are rare cases in abdominal wall surgery, with fewer than 300 cases reported up to the last major systematic review in 2019^[^2^]^. Their rarity, combined with a nonspecific clinical presentation, explains why they continue to be misdiagnosed as a lipoma, an abscess, a renal mass, or musculoskeletal pain, as reported in some published reports of delayed or incidental diagnosis^[^5,8,15^]^.

Diagnostic delay is not incidental. This is a consistently reported problem in the literature, as van Steensel *et al*^[^2^]^ reported that a considerable number of patients underwent imaging or even prior surgery for an alternative diagnosis before lumbar hernia was diagnosed. Lumbar hernia presenting as appendicitis within the hernial sac^[^7,16^]^, as in our first case, also demonstrates how misleading the initial clinical presentation can be. Our incidental case supports the recommendation that surgeons should inspect the posterolateral abdominal wall whenever diagnostic laparoscopy is undertaken for chronic, atypical abdominal pain.

Our second and third cases represent the classical and increasingly recognized entity of acquired lumbar hernia following retroperitoneal urological surgery. Nephrectomy and pyelolithotomy are well-documented but still underappreciated causes of late flank complications wherein division of the intercostal neurovascular bundles and denervation of the lateral abdominal musculature predispose to bulging and true herniation^[^12,17^]^.

The surgical approach to lumbar hernias has changed significantly away from open repairs. Historical descriptions have relied on open primary repair, autologous fascial or muscle flap reconstruction, and later open mesh repair; however, primary suture repair alone is associated with unacceptably high recurrence because the lumbar wall lacks a strong midline structure to bear tension, and the defect margins are often bony or muscular and friable^[^2^]^. The introduction of laparoscopic and extraperitoneal endoscopic mesh techniques has substantially improved outcomes by permitting tension-free repair with mesh overlap, while conferring the advantages of minimally invasive surgery, a finding supported by Park *et al*^[^6^]^, who noted shorter hospital stays for laparoscopic repairs than for open techniques.

Among the laparoscopic options, IPOM, TAPP, and TEP have each been described, with the choice largely dictated by defect size, prior scarring, and surgeon experience^[^10,11^]^. IPOM was our preferred technique in all three cases, as it avoids the extensive retroperitoneal or preperitoneal dissection required in TAPP or TEP. This is particularly advantageous in acquired hernias, in which previous surgical scarring limits tissue planes. This is consistent with the approach used by Miura *et al*^[^12^]^ in their report of a large acquired lumbar hernia and by Asami *et al*^[^18^]^ in a recent report combining IPOM with TAPP for simultaneous iatrogenic lumbar and inguinal hernia repair. Sharma *et al*^[^10^]^ described TEP repair, and Katsarelas *et al*^[^19^]^ described the TAPP approach with peritoneal flap creation for primary lumbar hernias, suggesting that this reduces direct mesh-bowel contact. Recently, retroperitoneal totally endoscopic approaches have been proposed by Li *et al*^[^20^]^ as a means of reducing the risk of intraperitoneal adhesions, although this technique demands a steep learning curve.

However, IPOM is not without risks. The placement of intraperitoneal mesh carries a risk of bowel adhesions, which can complicate future abdominal surgeries or lead to obstruction. However, the use of composite mesh in IPOM reduces the risk of bowel adhesions. Securing the mesh with tackers through the abdominal musculature can result in chronic, sharp abdominal wall pain. Other recognized limitations include seroma formation; the risk of mesh bulging or failure if the primary fascial defect is not adequately closed; and the complexity of managing intraperitoneal mesh infections if they arise.

Comparative data from these studies, together with retrospective series by Dasgupta *et al*^[^13^]^ and Dominguez-Bastante et al^[^9^]^, as well as studies by Heemskerk *et al*^[^1^]^ and a systematic review by van Steensel *et al*^[^2^]^, are summarized in Table 2. The postoperative hospital stay in most laparoscopic series is between 1 and 4 days, which aligns with our discharge on days 2–4. Reported recurrence rates after laparoscopic mesh repair of lumbar hernia are consistently low (0–5%) in the available literature, as compared with open suture repair series where recurrence has been 20–30%^[^1,2^]^. None of our three patients has developed recurrence, seroma, surgical site infection, or chronic pain on follow-up ranging from 6 months to 1 year.

**Table 2:** Summary of major studies on lumbar hernias.

Study (year)	Study type	*N* (cases)	Hernia etiology	Surgical approach	Key findings/complications
Asami *et al* (2026)^[18]^	Case report	1	Acquired (iatrogenic)	Combined TAPP + IPOM	4-day hospital stay; no short-term recurrence.
Dasgupta *et al* (2025)^[^13^]^	Retrospective	57	Mixed lateral hernias	Laparoscopic preperitoneal	2.64-day hospital stay; zero recurrences over 24 months.
Katsarelas *et al* (2025)^[19]^	Case report	1	Primary upper lumbar	Laparoscopic TAPP	Discharged on post-op day 1; excellent visualization.
Dominguez Bastante *et al* (2024)^[^9^]^	Case series	8	Mixed (primary/ acquired)	Lap/open mesh repair	Low complication rate and no recurrence.
Miura *et al* (2024)^[12]^	Case report	1	Acquired (post-nephrectomy)	Laparoscopic IPOM	4 days hospital stay; no recurrences.
Heemskerk *et al* (2023)^[^1^]^	Case series	6	Primary lumbar	TAPP	Proposes standardized algorithm to reduce variability.
Li H *et al* (2023)^[20]^	Case series	3	Mixed (primary/secondary)	Retroperitoneal endoscopic repair	2.3 days mean hospital stay; no short-term recurrence.
Li B *et al* (2021)^[^11^]^	Case series	10	Primary	Retroperitoneal TEP	1.5 days mean hospital stay; no recurrences.
van Steensel *et al* (2019)^[2]^	Systematic review	291	Primary lumbar	Various	Highlights 30.8% incarceration rate; 2% recurrence.
Park *et al* (2018)^[^6^]^	Retrospective	9	Secondary (traumatic)	Open/laparoscopic	Laparoscopy favored for hemodynamically stable patients.
Matsuda *et al* (2016)^[17]^	Case report	1	Acquired (post bone harvest)	Laparoscopic IPOM	6-day hospital stay. No recurrence.
Sharma *et al* (2005)^[^10^]^	Case report	1	Primary	Laparoscopic TEP	-

TAPP, transabdominal preperitoneal; IPOM, intraperitoneal onlay mesh; TEP, totally extraperitoneal.

## Limitations

This is a small case series of three patients, a mix of primary and acquired hernias. Furthermore, the absence of a comparator group and the relatively short follow-up period limit our ability to draw definitive conclusions regarding long-term recurrence rates compared to other surgical approaches. This small series cannot establish the superiority of IPOM over TAPP, TEP, or open repair; rather, it highlights IPOM as a viable option in surgical management.

## Conclusion

Lumbar hernias are a rare and often under-recognized cause of flank swelling or pain. A high index of clinical suspicion is required, particularly in patients with a history of unexplained lumbar pain or retroperitoneal surgery. Laparoscopic IPOM repair is a feasible, minimally invasive technique associated with favorable short- to intermediate-term outcomes, offering the benefits of full defect visualization and a short hospital stay.

## Data Availability

The data is available from the corresponding author on reasonable request.

## References

[1] Heemskerk J. Leijtens JWA, and van Steensel S. Primary lumbar hernia, review and proposals for a standardized treatment. J Abdom Wall Surg 2023;2:11754. 10.3389/jaws.2023.11754 PMC10831689 38312404

[2] van Steensel S Bloemen A van den Hil LCL. Pitfalls and clinical recommendations for the primary lumbar hernia based on a systematic review of the literature. Hernia 2019;23:107–17. 10.1007/s10029-018-1834-9 PMC6394702 30315438

[3] Suh Y Gandhi J Zaidi S. Lumbar hernia: a commonly misevaluated condition of the bilateral costoiliac spaces. Transl Res Anat 2017;8-9:1–5.

[4] Gonie Mekonnen A Bekele Gonfa K. Primary superior lumbar hernia: a rare cause of lumbar swelling. Int Med Case Rep J 2019;12:67–70. 10.2147/IMCRJ.S178727 PMC6404668 30881149

[5] Lillie GR Deppert E. Inferior lumbar triangle hernia as a rarely reported cause of low back pain: a report of 4 cases. J Chiropractic Med 2010;9:73–76. 10.1016/j.jcm.2010.02.001 PMC2943654 21629553

[6] Park Y Chung M Lee MA. Traumatic lumbar hernia: clinical features and management. Ann Surg Treat Res 2018;95:340–44. 10.4174/astr.2018.95.6.340 PMC6255748 30505826

[7] Ali SM Subramaniam S. Appendicular abscess as an unprecedented cause of an inferior lumbar hernia. Ann Royal Coll Surg Engl 2017;99:e85–e87. 10.1308/rcsann.2016.0351 PMC5392850 27869497

[8] Kadler B Shetye A Patten DK. A primary inferior lumbar hernia misdiagnosed as a lipoma. Ann Royal Coll Surg Engl 2019;101:e96–e98. 10.1308/rcsann.2019.0009 PMC6432969 30773901

[9] Dominguez Bastante M Fernandez Baeza M Santidrian Zurbano M. Lumbar hernia, a big unknown: case series on an experienced abdominal wall unit. Br J Surg 2024;111:znae122.283.

[10] Sharma A Panse R Khullar R. Laparoscopic transabdominal extraperitoneal repair of lumbar hernia. J Minim Access Surg 2005;1:70–73. 10.4103/0972-9941.16530 PMC3004108 21206649

[11] Li B Yu J Qin C. Retroperitoneal totally endoscopic prosthetic repair of primary lumbar hernia. Hernia 2021;25:1629–34. 10.1007/s10029-020-02334-z 33206280

[12] Miura T Tsujinaka S Nakano T. Laparoscopic intraperitoneal onlay mesh repair for symptomatic secondary lumbar hernia after retroperitoneoscopic nephrectomy: a case report. Cureus 2024;16:e75922. 10.7759/cureus.75922 PMC11658824 39703826

[13] Dasgupta P Ponnusamy R Pai A. Laparoscopic preperitoneal repair of lateral hernia - our experience. Mini-invasive Surg 2025;9:5.

[14] Agha RA Mathew G Rashid R. Revised preferred reporting of case series in surgery (PROCESS) guideline: an update for the age of Artificial Intelligence. Prem J Sci 2025;10:100080.

[15] Guennouni A Laridi A Abourak C. Lumbar hernia Jean-Louis Petit: a case report and literature review. Radiol Case Rep 2025;20:4579–82. 10.1016/j.radcr.2025.05.068 PMC12246593 40655300

[16] Arveen S Agila T. Acute appendicitis in an asymptomatic inferior lumbar hernia of Petit: a case report. Int J Abdom Wall Hernia Surg 2025;8:126–29.

[17] Matsuda A Miyashita M Matsumoto S. Laparoscopic mesh repair for lumbar hernia after iliac crest bone harvest. Asian J Endoscopic Surg 2016;9:314–17. 10.1111/ases.12308 27491627

[18] Asami Y Izumi H Ogasawara T. Simultaneous laparoscopic repair of iatrogenic lumbar and inguinal hernias using combined TAPP and IPOM: a case report. Tokai J Exp Clin Med 2026;51:64–68. 42366778

[19] Katsarelas I Chatzinas D Kountouri I. Laparoscopic transabdominal preperitoneal repair of a primary upper lumbar hernia: a case report. Cureus 2025;17:e79007. 10.7759/cureus.79007 PMC11911299 40099092

[20] Li H Cheng Z Yan W. Retroperitoneal totally endoscopic prosthetic repair of lumbar hernia. Sci Rep 2023;13:20736. 10.1038/s41598-023-48226-x PMC10676431 38007514

